# The 40S Ribosomal Protein S6 Response to Blue Light by Interaction with SjAUREO in *Saccharina japonica*

**DOI:** 10.3390/ijms20102414

**Published:** 2019-05-15

**Authors:** Hexiang Luan, Jianting Yao, Zhihang Chen, Delin Duan

**Affiliations:** 1 Key Laboratory of Experimental Marine Biology, Chinese Academy of Sciences, Qingdao 266071, China; luanhexiang87@163.com (H.L.); yaojianting@ms.qdio.ac.cn (J.Y.); kevinchenaddress@163.com (Z.C.); 2 Center for Ocean Mega-Science, Chinese Academy of Sciences, No.7 Nanhai Road, Qingdao 266071, China; 3 Laboratory for Marine Biology and Biotechnology, Qingdao National Laboratory for Marine Science and Technology, Qingdao 266237, China; 4 University of the Chinese Academy of Sciences, Beijing 100093, China

**Keywords:** *Saccharina japonica*, aureochrome, blue light, yeast two hybrid, 40S ribosomal protein S6

## Abstract

Blue light (BL) plays an important role in regulation of the growth and development of aquatic plants and land plants. Aureochrome (AUREO), the recent BL photoreceptor identified in photosynthetic stramenopile algae, is involved in the photomorphogenesis and early development of *Saccharina japonica* porophytes (kelp). However the factors that interact with the SjAUREO under BL conditions specifically are not clear. Here in our study, three high quality cDNA libraries with CFU over 5 × 10^6^ and a recombination rate of 100% were constructed respectively through white light (WL), BL and darkness (DK) treatments to the juvenile sporophytes. Based on the constructed cDNA libraries, the interactors of SjAUREO were screened and analyzed. There are eighty-four genes encoding the sixteen predicted proteins from the BL cDNA library, sixty-eight genes encoding eighteen predicted proteins from the DK cDNA library, and seventy-four genes encoding nineteen proteins from the WL cDNA library. All the predicted proteins are presumed to interact with SjAUREO when co-expressed with SjAUREO seperately. The 40S ribosomal protein S6 (RPS6), which only exists in the BL treated cDNA library except for two other libraries, and which is essential for cell proliferation and is involved in cell cycle progression, was selected for detailed analysis. We showed that its transcription was up-regulated by BL, and was highly transcribed in the basal blade (meristem region) of juvenile sporophytes but less in the distal part. Taken together, our results indicated that RPS6 was highly involved in BL-mediated kelp cellular division and photomorphogenesis by interacting with SjAUREO.

## 1. Introduction

*Saccharina japonica* is one of the most important commercial brown seaweeds, and it could be used for food, alginate [1,2] and polysaccharides [3] extraction. With the characteristic of inching in the lower coastal environments, it utilizes specific wavelengths of the solar spectrum [4] for growth and development. Among the different light wavelengths, blue light which falls in the short wavelength region is predominant under the sea [5,6,7]. Photoreceptors are important proteins in most plants that are useful for adapting their physiology to the environmental light conditions, which can perceive and transfer light information to the downstream components [8,9]. There are at least four distinct families of photoreceptors that have been found to function in light perception, which include cryptochromes, phytochromes, phototropins and ultraviolet B (UVB) photoreceptor(s). Plants require the UV-B photoreceptor UV resistance locus 8 (UVR8) for acclimation and survival under the sun. Upon UV-B perception, UVR8 switches instantaneously from a homodimeric to monomeric configuration, which leads to the interaction with the key signaling protein constitutively photomorphogenic 1 (COP1) and induction of UV-B–protective responses [10]. Phytochromes are reversible between red/far-red light-activated molecular forms which particulate the regulation of both physiological and developmental responses, such as seed germination, photomorphogenesis and chloroplast movement, shade avoidance, and circadian rhythm [11].

Photoreceptors mediating the regulation of metabolic processes using blue light were determined to be cryptochrome (CRY) and phototropin (PHOT). CRYs are far less widespread and are found in higher plants and most animals, but only in a handful of other eukaryotes and prokaryotes sharing a common evolutionary ancestor with DNA photolyases [12]. All of the CRY proteins have a conserved photolyase homology region (PHR) of about 500 amino acids and a C-terminal extension of varying lengths. In the PHR domain, flavin adenine dinucleotide (FAD) is bound as a chromophore to control different biological processes [13]. CRYs are derived from the photolyases that catalyze the blue-light dependent repair of UV light-induced damages [14]. The PHOT proteins are plasma membrane localized protein kinases and mediate phototropism responses in the entire green lineage, which contains an N-terminal photosensory region, a C-terminal AGC-type Ser/Thr protein kinase domain and two light oxygen voltage (LOV1 and LOV2) domains [13]. Blue light is sensed by two flavin mononucleotide (FMN) LOV1 and LOV2 domains [15]. In the dark, LOV2 binds to the kinase domain and inhibits its phosphorylation activity. Light inhibits the binding between the kinase domain and LOV2, resulting in the activation of kinase activity. Upon blue-light irradiation, PHOT1 moves rapidly to the cytoplasm, while a fraction of PHOT2 moves to the Golgi apparatus [16].

During the course of evolution through various endosymbiotic processes, diverse photosynthetic eukaryotes acquired BL responses that do not use photosynthetic pathways. Photosynthetic stramenopiles, which received chloroplasts from the red algae during the secondary endosymbiosis, are phylogenetically different from green plants [17]. Photosynthetic stramenopile BL receptor aureochrome (AUREO) was undefined until its discovery in 2007 from the photosynthetic stramenopile alga, Vaucheria [18]. Aureochromes, a kind of BL receptor different from cryptochrome and phototropin, followed by discovered in yellow-green alga *Chattonella antiqua* [19], *Saccharina japonica* [20] and *Phaeodactylum tricornutum* [21,22], and therefore also represents a BL receptor in photosynthetic stramenopiles [7,23]. So far, there are no reports of the presence of aureochromes in green algae. Structurally, aureochromes harbor two conserved domains: A light-oxygen-voltage (LOV) domain at the N-terminus for light reception as well as a basic region leucine zipper (bZIP) domain within the C-terminal region for DNA binding, which has been suggested to function as a light regulated transcription factor [18,24,25,26]. Originally, two Aureo homologs, named Aureo1 and 2, were identified, but only Aureo1 was shown to bind DNA in a light-dependent manner [11]. The light-sensing and DNA-binding parts of AUREO were arranged in a different way to the arrangement seen in most related photoreceptors [27]. In the dark, the LOV domain directly interacts with the bZIP domain and thereby impedes its DNA binding function while illumination with blue light triggers intramolecular bZIP-LOV dissociation and subsequent LOV dimerization, thus enhancing the affinity of PtAu1a for its target DNA sequence [28]. In *Phaeodactylum tricornutum*, Aureo may participate in the control of G1/S transition in the cell cycle of diatoms [29].

Previously, we studied the association of SjAUREO with BL-mediated photomorphogenesis during kelp growth and early development [20], however, the factors that specifically interact with SjAUREO under BL remain unknown. In order to find novel interaction partners of SjAUREO, we applied SjAUREO as bait for screening its interactors from WL, BL and DK treated cDNA libraries to identify the interactors that uniquely experienced a response to BL. It is expected that the mechanisms of BL mediated cellular division and early development in the *S. japonica* will be explored.

## 2. Results

### 2.1. cDNA Three-Frame Library Construction and Evaluation

With the illumination of BL, WL and DK to *S. japonica* juvenile sporophytes, the treated kelp materials were collected and then were applied for the library construction. The constructed three-frame cDNA library plasmids were mixed and transformed into *Escherichia coli* DH10B cells. The three cDNA libraries were estimated by calculating the CFU (colony-forming units) and recombination rate (RR). Table 1 showed that each reading frame of the library had >7 × 10^6^ primary clones thereby ensuring a complete representation of rare sequences. Twenty-four positive clones were randomly selected from each three primary libraries and identified by PCR. All the twenty-four colonies showed bands with an average insert size of 0.5~4.2 kb (Supplemental Figure S1), indicating a 100% recombination rate. 

### 2.2. The Bait Construction, Toxicity and Auto-Activation Validation

**Figure 1:**
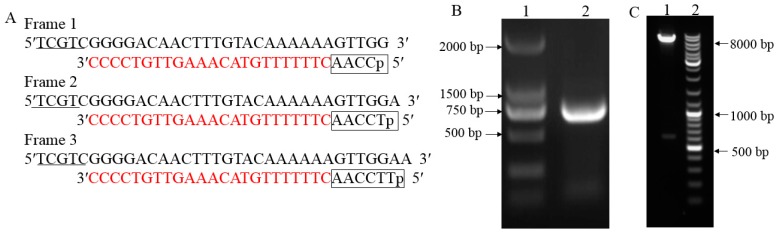
The three reading frames pGADT7 attB vector reconstruction and bait vector construction. (**A**): Reconstruction of pGADT7 attB vector for the generation of three reading frames. pGADT7 vector was reconstructed to represent the three possible reading frames by inserting one (T) or two nucleotides (TT) behind the attB1 sequence with AACC which is boxed. The 5′ end contains protective bases that are underlined. (**B**): Gel electrophoresis image of aureochrome (AUREO) amplification from cDNA. Lane 1 is DNA marker whose strip size is writen on the left, lane 2 is the PCR product of AUREO from cDNA. (**C**): Gel electrophoresis image of the recombinant plasmid AUREO-pGBKT7 which was digested by endonuclease *Nde* I and *Sal* I. Lane 1: AUREO-pGBKT7 digested by *Nde* I and *Sal* I; lane 2: DNA marker whose strip size is indicated on the right.

To identify protein interaction with SjAUREO, we cloned open reading frames (ORFs) from the *S. japonica* into the yeast two-hybrid bait vector for expression of the proteins with an N-terminal GAL4 DNA binding domain (DBD). The coding sequences (CDS) with the addition of the *Nde* I and *Sal* I sites were amplified from the *S. japonicac* DNA with a fragment length of 609 bp (Figure 1B). The fragment was introduced into the bait vector by the enzyme linked method to generate the bait construct. After digesting the product by *Nde* I and *Sal* I, two fragments as expected with length of about 7.3 kb and 0.7 kb were released (Figure 1C). Both PCR product as well as the bait construct were sequenced and the results showed they were 100% homologous with the *SjAUREO* from *S. japonicac* genome (Supplemental Figure S2), indicating that the bait construct was made successful and ready for yeast two hybrid assay. In order to check the toxicity and auto-activation of SjAUREO protein in yeast, the bait plasmid was transformed alone and co-transformed with the prey plasmids pGADT7, respectively. After a four day incubation at 30 °C, the AUREO-pGBKT7 transformants grew well on the SD^-Le^^u^ plate and could not grow on the SD^-Leu-Trp^ and SD^-Leu-Trp-His-Ade^ plates, which indicated that the SjAUREO could tolerate the absence of leucine (Leu) as the pGBKT7 contained this amino acid marker, but they were tryptophan auxotrophs and could not tolerate the media without tryptophan (Trp), or activate the reporter gene histidine (His) and adenine (Ade) by itself. Approximately 100–1000 colonies of the co-transformation appeared on each SD^-Leu-Trp^ plate, with no significant growth on SD^-Leu-Trp-His-Ade^ plate, which indicated that as the transformant contained both bait and prey vectors they could not interact with each other, resulting in no activation of the His and Ade reporter gene expression. Generally, the AUREO-pGBKT7 could not activate the reporter genes and could be used for library screening.

### 2.3. Searching for SjAUREO Interactors

The Y2HGold cell expressing the bait protein SjAUREO was used for preparing the competent cells, and transformed with each yeast expression library for screening separately. The total number of transformants obtained was: DK group 2.16 × 10^7^, BL group 2 × 10^7^ and WL group 2.8 × 10^7^, and all the transformants were over to 8 × 10^5^ for the following assay. The positive interactors which grew well on the SD^-^^Leu-^^Trp-^^His-^^Ade^ plates were picked randomly for sequence analysis and function prediction. The BLAST tools were used to identify presumptive orthologs. A total of 68 cDNA sequences were obtained from the DK library and were annotated for the presumptive function, and only 34 sequences were predicted with 18 functional proteins (Table 2), while the others were unknown. A total of 84 cDNA sequences were blasted from BL library, and only 28 sequences were annotated with 16 different functional proteins (Table 3), while the remaining 56 sequences were unknown. The WL library group showed 74 colonies to be sequenced while 48 were unknown, and 26 sequences were annotated into 19 proteins (Table 4). Among these annotated function proteins, light harvesting complex protein (LHC) and sporulation-like protein (SPLP) were detected in BL, WL and DK libraries.

**Figure 2:**
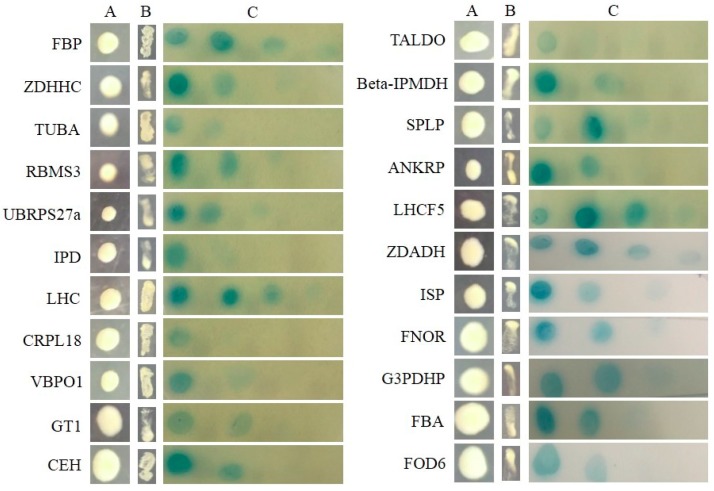
Plasmid linkage assays for DK transformants identified during yeast two-hybrid screening. Transformants containing the bait and prey plasmids were identified based on their growth on dropout medium (SD^-Leu-Trp^; column A). Putative interacting partners were identified based on their growth on SD^-Leu-Trp-His-Ade^ (column B), and the interaction strength was detected on SD^-Leu-Trp-His-Ade^ medium with α-x-gal (column C), each drop concentration ranged from 10^−1^, 10^−2^, 10^−3^ to 10^−4^.

**Figure 3:**
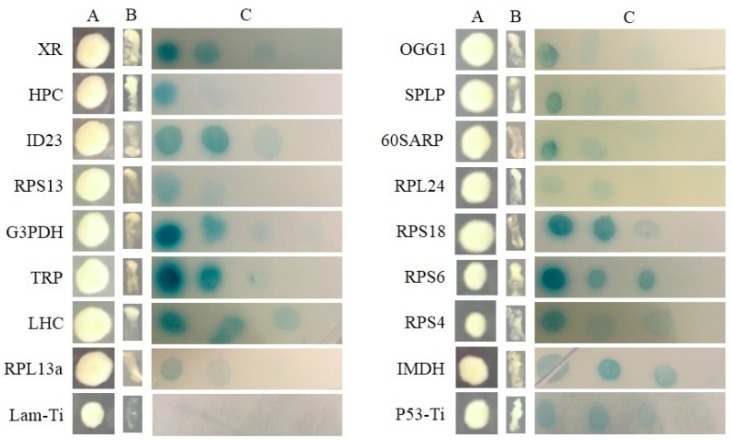
Plasmid linkage assays for BL transformants identified during yeast two-hybrid screening. Transformants containing the bait and prey plasmids were identified based on their growth on dropout medium (SD^-Leu-Trp^; column A). Putative interacting partners were identified based on their growth on SD^-Leu-Trp-His-Ade^ (column B), and the interaction strength was detected on SD^-Leu-Trp-His-Ade^ medium with α-x-gal (column C), each drop concentration ranged from 10^−1^, 10^−2^, 10^−3^ to 10^−4^. pGBKT7-53 + pGADT7-Ti was used as positive control, while pGBKT7-Lam + pGADT7-Ti served as negative control.

**Figure 4:**
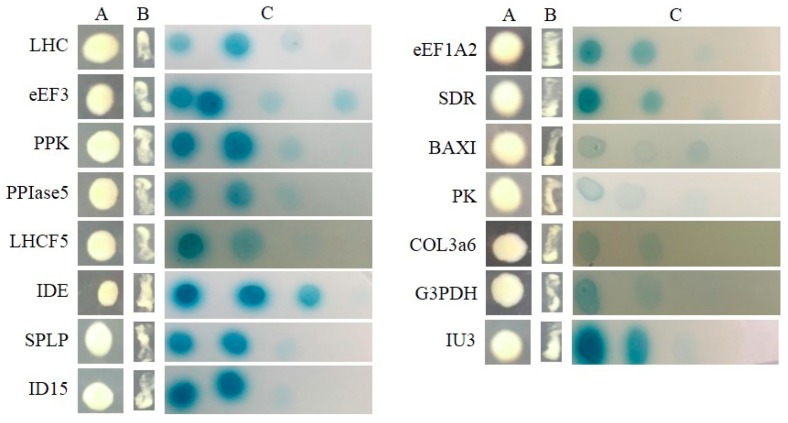
Plasmid linkage assays for WL transformants identified during yeast two-hybrid screening. Transformants containing the bait and prey plasmids were identified based on their growth on dropout medium (SD^-Leu-Trp^; column A). Putative interacting partners were identified based on their growth on SD^-Leu-Trp-His-Ade^ (column B), and the interaction strength was detected based on the growth on SD^-Leu-Trp-His-Ade^ medium with α-x-gal (column C), each drop concentration ranged from 10^−1^, 10^−2^, 10^−3^ to 10^−4^.

All of the interactions from three libraries were confirmed using the re-transformation assay. The known function interactors were isolated and co-transformated with SjAUREO and selected in the SD^-^^Leu-^^Trp-^^His-^^Ade^ media and by their ability to express beta-galactosidase protein using an agarose overlay α-x-gal assay (Figure 2, Figure 3 and Figure 4). The *lacZ* reporter was used to assess the strength of interaction of the individual transformants selected in a library screen. Additionally, the yeast cells co-transformed with pGBKT7-Lam and pGADT7-T were taken as a negative control, whereas pGBKT7-53 and pGADT7-T served as a positive control. The interaction intensity was also surveyed by different concentration (from 10^−1^, 10^−2^, 10^−3^, 10^−4^) drops on SD^-^^Leu-^^Trp-^^His-^^Ade^ with α-x-gal. The fructose 1,6-bisphosphatase (FBP), light harvesting complex protein (LHC), chloroplast light harvesting protein lhcf5 (LHCF5) and Zn-dependent alcohol dehydrogenase family (ZDADH) had the strongest interactions with SjAUREO at the DK library group (Figure 2). Among the 16 partners from the BL library, imm downregulated 23 (ID 23), LHC, RPS6, and 3-isopropylmalate dehydrogenase (IMDH) showed high affinity to SjAUREO (Figure 3). While in the WL group, elongation factor EF-3 (eEF3), phosphoglycerate kinase (PPK) and insulin-degrading enzyme (IDE) indicated strong interaction with SjAUREO (Figure 4). The gene names for each acronym are indicated in Table 2, Table 3 and Table 4.

### 2.4. Bimolecular Fuorescence Complementation (BiFC) Assay and Expression Analyses of 40S Ribosome S6 Gene Detected with qRT-PCR

**Figure 5:**
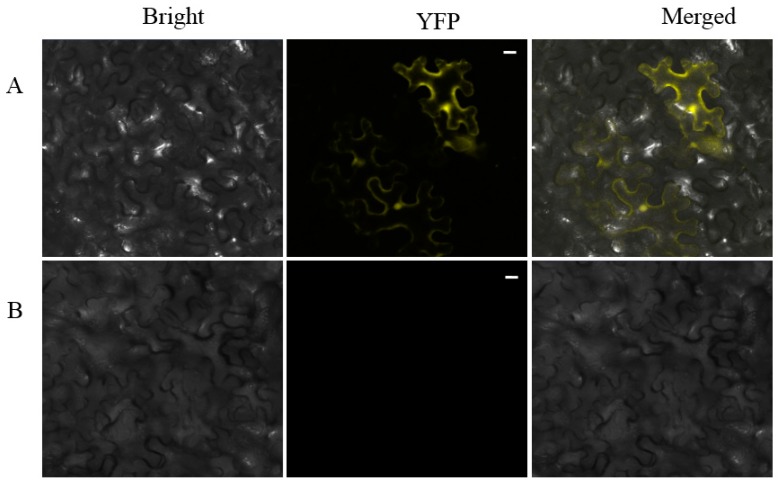
Bimolecular fluorescence complementation (BiFC) assay demonstrating the interaction between SjAUREO and RPS6 in planta. 40X magnification of micrographs at 48 h post-infiltration from plants co-expressing nEYFP-SjAUREO and cEYFP-RPS6 (**A**), with nEYFP and cEYFP (**B**) are served as negative control, are shown in *N. benthamiana*. Images are representative of three separate infiltrations from two independent experiments. Scale bars: 20 µm.

After screening the three light treated libraries, we wanted to determine which genes responded to BL specifically. The 40s ribosomal proteinS6 (RPS6) which was detected only in the BL library and showed strong affinity to SjAUREO (Figure 3) and appeared three times (Table 3) indicating its importance in the progress of SjAUREO function, was then selected for further characterization. To further confirm the interaction between SjARUEO and RPS6, the BiFC assay was introduced in epidermal cells. Results showed that the co-infiltrations of nEYFP-SjAUREO and cEYFP-RPS6 produced strong fluorescence signals in *N. benthamiana* cells (Figure 5A), while no fluorescence signal was observed for the negative control nEYFP and cEYFP (Figure 5B). These results confirmed that SjARUEO interacts with RPS6s in living plant cells. 

**Figure 6:**
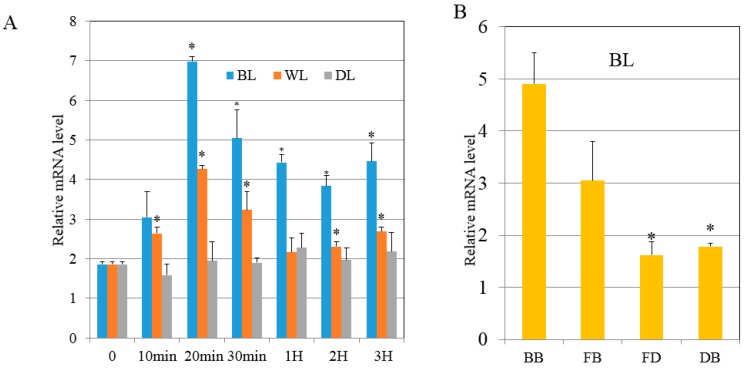
Expression profiling of RPS6. (**A**): The relative mRNA level of RPS6 during the time course at each light condition. (**B**): The relative mRNA level of RPS6 at different position of alga exposed to BL. BB (basal blade), FB (frond near base blade), FD (frond near distal blade) and DB (distal blade). Results are representative of three independent repeats. Error bars indicate SD (*n* = 3). Asterisks denote significant difference from 0 time point as determined by *t* test.

Previous research showed that *RPS6* had a role in in cell division [30]. There was an increase in *SjRPS6* transcription which was highest after 20 min under BL illumination after preculturing in the DK (Figure 6A). While in WL, lower *SjRPS6* transcription occurred after preculturing in the DK (Figure 6A). This implied that the *SjRPS6* induction was specific to blue light. To further verify the *SjRPS6* transcripts relation with the kelp parts, we applied different parts of the juvenile sporophytes (BB, FB, FD and DB) and exposed them to BL for 20 min, qRT-PCR analysis was also done. The results indicated that the *SjRPS6* expression was higher at the BB and decreased from BB to the DB regions (Figure 6B).

## 3. Discussion

The yeast two-hybrid screen is a widely used approach for screening the binding/interacting proteins to the objective proteins [31]. Here, three full-length three-frame cDNA libraries from the BL, DK and WL treated juvenile sporophytes respectively were constructed and applied to yeast two-hybrid system. For the positive interactors screening, the library was screened intensively to maximize the chance of capturing related proteins. To the three possible reading frames which was constructed by inserting one (T) or two nucleotides (TT) behind the attB1 sequence, we enriched the cDNA library for in-frame activation domain fusions and to improve the success ratios of objective protein screening (Figure 1A). According to the high-efficiency protocol, the yield transformation efficiencies should be within the range of 5 × 10^5^ to 2 × 10^6^ cfu to guarantee covering the cDNA library at least 2–3 times [32]. For each amplified three-frame cDNA library containing >7 × 10^6^ cfu (Table 1), the parameters were 0.5~4.2 kb (average insertion size) and 100% recombination rate, which indicated that high-quality cDNA libraries were constructed and could be used further.

In this study, we used SjAUREO as bait to screen the algae cDNA library from BL, WL and DK for proteins responding only to BL. Among the interacting partners of SjAUREO, the SjRPS6 (Table 3) was chosen for further investigation because of its strong affinity (Figure 3) and role in cell division. Theoretically, ribosome biogenesis is essential for cell proliferation [30]. The phosphorylation of RPS6 might be directly associated with regulating the cell size [33,34] and was most highly stimulated by the light signal in plant [35]. Judging from our data, the increase of *SjRPS6* transcriptions was significantly higher when exposed to the blue light condition (Figure 5A) and most abundant in the basal blade (Figure 5B), and this implied that this part was more sensitive than the other parts of kelp.

The process of how SjAUREO activated the transcription of PRS6 remains to be elucidated. Previous documents hypothesized that the LOV domain in SjAUREO changed after BL illumination, inducing a conformational change in the homodimer SjAUREO complex and activating the initiation of BL signal transduction through interaction of bZIP domain with different regulatory elements [13]. Here we deduced that PRS6 could interact with SjAUREO via its bZIP domain. Nevertheless, both in vivo and in vitro studies are required to prove these presumptions.

## 4. Materials and Methods

### 4.1. Kelp Materials

Fresh juvenile sporophytes of *S. japonica* were collected from the cultivated rafts in Rongcheng, China in Dec, 2016. The robust samples were selected and rinsed with sterilized seawater at least three times to remove epiphytes, and pre-cultured in constant darkness for 12 h at 10 °C. All the samples were divided into three equal parts, transferred and immersed to new sterilized seawater under BL, DK, and WL for 3 h respectively. Blue light conditions were set as described previously [13] and white light was set up as 100 μmol photons m^−^^2^ s^−^^1^ by a quantum photometer. The kelp samples were collected, dried with hygroscopic filter paper, and dissected into four parts: Basal blade (BB), frond near base blade (FB), frond near distal blade (FD) and distal blade (DB). All the kelp materials were frozen immediately in liquid nitrogen for the following RNA extraction. Three independent biological replicates were performed.

### 4.2. RNA Extraction and Quantitative RT-PCR Analysis

The total RNA of the whole and different parts of *S. japonica* were extracted by the CTAB method as described by Yao et al. [36]. Reverse transcription (RT) and first-strand cDNA synthesis were carried out according to the previous research [37]. Three independent RNA preparations were analyzed by quantitative RT-PCR (qRT-PCR) to evaluate relative differences in transcript levels. Primers (qRPS6 F: AGGAGGTGGATGGCGAGGCT, qRPS6 R: TTCTTGGTGACGACGAGGTT) were designed to amplify genes of about 200 bp in size. Transcript levels for the target gene were normalized to the endogenous β-actin transcripts from *S. japonica* as an internal control using primer actin F: AGGAGACGGGTAAGGAAGAA and actin R: GTGGTCATGCTACTACACACA. qRT-PCR was carried out in 96 well plates using SYBR Green-Mix, with cycling conditions set to 95 °C for 1 min, 40 cycles of 95 °C for 10 s, and 60 °C for 30 s. Gene expression was quantized using the relative quantification (2^-ΔΔCt^) method. The P value of <0.01 was considered sufficient for rejection of the null hypothesis. Each sample or treatment was tested in at least three biological repeats and the same experiment was performed twice.

### 4.3. cDNA Three-Frame Library Construction and Evaluation

In order to have high quality cDNA library, the Gateway system was introduced. The mRNA from DK, BL and WL treatment were isolated and ligated with three different adapters with attB1 sequences (Figure 1A). The three-frame cDNA libraries were generated by recombination through the attB sites and mixed equally and electro-transformed into *E. coli* DH10B cells for amplification. The amplified cDNA library plasmid was transformed to Y2HGold yeast strain (Clontech Laboratories, Dalian, China) competent cells, and the transformation efficiency as well as library titer were calculated to make sure the cDNA library was ready for use [38]. Briefly, the transformed yeast was diluted 1000 times and 50 μL from the 2 mL was used for plating. The colonies were counted and total CFU (colony-forming units) was calculated by CFU = colonies number/50 μL × 1000 × 1000 μL × 2 mL. Twenty-four positive clones were randomly selected from each library and identified by PCR to calculate the recombination rate.

### 4.4. The Prey Vector Construction and Application to Y2H

With the identified information of full-length SjAUREO protein in our previous research [13], the SjAUREO was amplified from the *S. japonica* cDNA by using forward (CAAGAGGCCATTACGGCCATGTCGGAGCAGCAGAAGCTGG) and reverse (AACGGATCCCCGCAGCCCCTGTACGGCAACGAAAAAC) primers containing the *Sfi* I and *BamH* I restriction sites. The SjAUREO bait vector (pGBKT7-AUREO) was constructed using the appropriate restriction enzymes (TaKaRa Bio Inc., Otsu, Japan), in order to clone the *SjAUREO* gene into pGBKT7 (Clontech Laboratories, Dalian, China). The pGBKT7-AUREO was sequenced prior to transformation to ensure that the bait protein (SjAUREO) encodes the correct reading frame of the GAL4 DNA binding domain. The fused construct pGBKT7-AUREO was subsequently transformed into the Y2HGold yeast strain according to the user manual of the Yeast-maker™ Yeast Transformation System 2 (Clontech Laboratories, Dalian, China). The toxicity and auto-activation of bait protein were examined according to the user manual of the Matchmaker™ Gold Yeast Two-Hybrid System (Clontech Laboratories, Dalian, China).

### 4.5. Yeast Two-Hybrid Library Screening and Interaction Conformation

The Y2HGold harboring the pGBKT7-AUREO reporter plasmid was transformed with plasmid from three different libraries. The transformants were placed on agar plates with synthetic media containing dextrose lacking leucine (Leu) and tryptophan (Trp) and incubated for two days at 30 °C. The surviving transformants were streaked on plates containing galactose and 5-bromo-4-chloro-3-indolyl-a-d-galactopyranoside without Leu, Trp, His and Ade, and incubated for three days at 30 °C. All positive prey plasmids were sent for DNA sequencing by the sequencing primer in the pGADT7 vector and blasted against NCBI database. The two-hybrid interaction was confirmed by retransformation of the yeast strain Y2HGold with the bait vector and purified prey plasmid followed by streaking on selection plates SD^-Leu-Trp-His-Ade^ and plates with 5-bromo-4-chloro-3-indolyl-a-d-galactopyranoside.

### 4.6. Bimolecular Fluorescence Complementation Assays

For BiFC assays, the interaction proteins SjAUREO and RPS6 were cloned to n/cEYFP vectors and transformed into the *Agrobacterium tumefaciens* strain EHA105. The nEYFP and cEYFP served as negative controls. Positive agrobacteria which fused with reciprocal halves of EYFP were co-infiltrated into *N. benthamiana* plants. Leaf tissues descended in water drop reversal after two days and were checked by confocal microscopy of PLAPO60XWLSM (NA 1.0) objective. The interaction was representative of three separate infiltrations from two independent experiments.

## 5. Conclusions

Three high quality three-frame cDNA libraries of *Saccharina japonica* with CFU over 5 × 10^6^ and recombination rate of 100% were constructed by Gateway system. Interactors of SjAUREO from WL, BL and DK treated library were analyzed online for predicated functions. The RPS6 interacted with SjAUREO especially under BL condition was up-regulated and highly transcribed in the basal blade indicated its role in BL-mediated kelp cellular division and photomorphogenesis.

## Figures and Tables

**Table 1:** Results for the cDNA library quality identification.

Group	CFU	RR
DK	8.4 × 10^6^	100%
BL	1.2 × 10^7^	100%
WL	7.8 × 10^6^	100%

DK: Dark, BL: Blue light, WL: White light, RR: recombination rate.

**Table 2:** BLAST analysis of yeast two-hybrid results using SjAUREO as bait to screen the DK treated cDNA library.

Accession No.	Characteristics	Homology	Clone Number
CBN75354.1	Fructose 1,6-bisphosphatase (FBP)	54%	2
CBJ48329.1	Fructose-1,6-bisphosphate aldolase (FBA)	96%	1
CAQ51444.1	Putative vanadium-dependent bromoperoxidase 6 (VBP 6)	55%	1
CAD37191.1	Vanadium-dependent bromoperoxidase 1 (VBP 1)	64%	1
EJY76073.1	DHHC zinc finger domain containing protein (ZDHHC)	65%	1
CBJ28184.1	Tubulin alpha (TUBA)	76%	1
KKF25031.1	RNA-binding motif,Single-stranded-interacting protein 3-like (RBMS3)	97%	2
CBN74156.1	Similar to ubiquitin and ribosomal protein S27a precursor (UBRPS27a)	86%	1
OPX53870.1	3-isopropylmalate dehydrogenase (IMDH)	99%	3
CBJ25909.1	Cytoplasmic ribosomal protein L18 (CRPL18)	95%	1
CBN77006.1	Light harvesting complex protein (LHC)	75%	1
ACE80197.1	Chloroplast light harvesting protein lhcf5 (LHCF5)	100%	2
CBN75623.1	Phosphoglycerate kinase (PGK)	93%	1
CAD37191.1	Vanadium-dependent bromoperoxidase 1 (VBPO1)	89%	2
WP_026719310.1	Glycosyltransferase family 1 protein (GT1)	46%	1
CBJ31450.1	CAB/ELIP/HLIP superfamily protein (CEH)	86%	3
WP_094674345.1	Transaldolase (TALDO)	45%	1
AAA66917.1	Beta-isopropylmalate dehydrogenase (Beta-IPMDH)	95%	1
ABP93411.1	Sporulation-like protein (SPLP)	90%	1
CBJ30026.1	Ankyrin repeat protein (ANKRP)	46%	1
EGA60350.1	Zn-dependent alcohol dehydrogenase family (ZDADH)	51%	1
CBJ29582.1	Cytochrome b6-f complex iron-sulfur subunit (ISP)	83%	1
ABU96658.1	glyceraldehyde-3-phosphate dehydrogenase precursor (G3PDHP)	99%	1
CBN78345.1	Ferredoxin-NADP oxidoreductase (FNOR)	86%	1
CBJ31978.1	Flagellar outer dynein arm light chain 6 (FOD6)	99%	1

**Table 3:** BLAST analysis of yeast two-hybrid results using SjAUREO as bait to screen the BL treated cDNA library.

Accession No.	Characteristics	Homology	Clone Number
EWM23897.1	40S ribosomal protein S18 (RPS18)	74%	1
EWM21878.1	40S ribosomal protein S6 (RPS6)	75%	3
CBJ29820.1	40S ribosomal protein S4 (RPS4)	87%	1
OPX53870.1	3-isopropylmalate dehydrogenase (IMDH)	95%	4
CBJ28016.1	Similar to L-xylulose reductase (XR)	94%	1
XP_005844401.1	Hypothetical protein chlncdraft_139059 (HPC)	68%	3
CBJ27783.1	Imm downregulated 23 (ID 23)	72%	1
ACN09872.1	40S ribosomal protein S13 (RPS13)	79%	1
CBJ31240.1	Chloroplast glyceraldehyde-3-phosphate dehydrogenase (G3PDH)	83%	1
CBJ31434.1	Transient receptor potential channel fragment (TRP)	63%	1
CBN78494.1	Light harvesting complex protein (LHC)	89%	2
CBJ25662.1	60s ribosomal protein L13a (RPL13a)	91%	4
CBJ27158.1	Endonuclease III/similar to 8-oxoguanine DNA glycosylase isoform 1b (OGG1)	60%	1
ABP93411.1	Sporulation-like protein (SPLP)	96%	2
EWM29947.1	60s acidic ribosomal protein (60SARP)	50%	1
CBJ28054.1	Ribosomal protein l24 (RPL24)	89%	1

**Table 4:** BLAST analysis of yeast two-hybrid results using SjAUREO as bait to screen the WL treated cDNA library.

Accession No.	Characteristics	Homology	Clone Number
CBJ33313.1	Light harvesting complex protein (LHC)	83%	3
CBJ25582.1	Elongation factor EF-3 (eEF3)	94%	1
CBN75623.1	Phosphoglycerate kinase (PPK)	93%	3
CBJ26174.1	FKBP-type peptidyl-prolyl cis-trans isomerase 5 (PPIase5)	76%	1
ACE80197.1	Chloroplast light harvesting protein lhcf5 (LHCF5)	100%	6
CBN77215.1	insulin-degrading enzyme (IDE)	69%	1
ABP93411.1	Sporulation-like protein (SPLP)	99%	1
CBJ25754.1	Imm downregulated 15 (ID15)	87%	1
CBN74763.1	Imm upregulated 3 (IU3)	63%	1
CBJ31240.1	Glyceraldehyde 3-phosphate dehydrogenase (G3PDH)	71%	1
CBJ32893.1	eukaryotic translation elongation factor 1 alpha (eEF1A2)	93%	3
CBN74950.1	Short-chain dehydrogenase/reductase (SDR)	56%	1
CBN79712.1	Putative BAX inhibitor (BAXI)	81%	1
AIT70004.1	Pyruvate kinase (PK)	77%	1
XP_014788837.1	collagen alpha-3(VI) chain-like (COL3a6)	29%	1

## Supplementary Materials

Supplementary materials can be found at https://www.mdpi.com/1422-0067/20/10/2414/s1.

## References

[1] Hay I.D., Rehman Z.U., Moradali M.F., Wang Y., Rehm B.H.A. (2013). Microbial alginate production, modification and its applications. Microb. Biotechnol..

[2] Zhang P., Shao Z., Jin W., Duan D. (2016). Comparative characterization of two GDP-mannose dehydrogenase genes from *Saccharina japonica* (Laminariales, Phaeophyceae). BMC Plant. Bio..

[3] Vishchuk O.S., Ermakova S.P., Zvyagintseva T.N. (2011). Sulfated polysaccharides from brown seaweeds *Saccharina japonica* and *Undariapinnatifida*: Isolation, structural characteristics, and antitumor activity. Carbohydrate Res..

[4] Hegemann P. (2008). Algal sensory photoreceptors. Annual Rev. Plant Biol..

[5] Ragni M., d’Alcalà M.R. (2007). Circadian variability in the photobiology of *Phaeodactylumtricornutum*: Pigment content. J. Plankton Res..

[6] Depauw F.A., Rogato A., Ribera d’Alcalá M., Falciatore A. (2012). Exploring the molecular basis of responses to light in marine diatoms. J. Exp. Bot..

[7] Matiiv A.B., Chekunova E.M. (2018). Aureochromes–Blue Light Receptors. Biochemistry.

[8] Jiao Y., Lau O.S., Deng X.W. (2007). Light-regulated transcriptional networks in higher plants. Nat. Rev. Genet..

[9] Masuda S., Hasegawa K., Ohta H., Ono T.A. (2008). Crucial role in light signal transduction for the conserved Met93 of the BLUF protein PixD/Slr1694. Plant Cell Physiol..

[10] Rizzini L., Favory J.J., Cloix C., Faggionato D., O”Hara A., Kaiserli E., Baumeister R., Schäfer E., Nagy F., Jenkins G.I. (2011). Perception of uv-b by the arabidopsis uvr8 protein. Science..

[11] Sharrock R.A. (2008). The phytochrome red/far-red photoreceptor superfamily. Genome Bio..

[12] Chaves I., Pokorny R., Byrdin M., Hoang N., Ritz T., Brettel K., Essen L.O., van der Horst G.T., Batschauer A., Ahmad M. (2011). The cryptochromes: Blue light photoreceptors in plants and animals. Annu. Rev. Plant Biol..

[13] Galvão V.C., Fankhauser C. (2015). Sensing the light environment in plants: Photoreceptors and early signaling steps. Curr. Opin. Neurobiol..

[14] Kottke T., Oldemeyer S., Wenzel S., Zou Y., Mittag M. (2017). Cryptochrome photoreceptors in green algae: Unexpected versatility of mechanisms and functions. J. Plant Physiol..

[15] Suetsugu N., Wada M. (2013). Evolution of three lov blue light receptor families in green plants and photosynthetic stramenopiles: Phototropin, ztl/fkf1/lkp2 and aureochrome. Plant & Cell Physiol..

[16] Jeong R.D., Chandra A.C., Barman S.R., Navarre D., Klessig D.F., Kachroo A., Kachroo P. (2010). Cryptochrome 2 and phototropin 2 regulate resistance protein-mediated viral defense by negatively regulating an E3 ubiquitin ligase. Proc. Natl. Acad. Sci. USA.

[17] Cavalier S.T., Robertson D.L. (1986). The kingdom chromista origin and systemmatics. Progr. Phycol. Res..

[18] Takahashi F., Yamagata D., Ishikawa M., Fukamatsu Y., Ogura Y., Kasahara M., Kiyosue T., Kikuyama M., Wada M., Kataoka H. (2007). Aureochrome, a photoreceptor required for photomorphogenesis in stramenopiles. Proc. Natl. Acad. Sci. USA.

[19] Ishikawa M., Takahashi F., Nozaki H., Nagasato C., Motomura T., Kataoka H. (2009). Distribution and phylogeny of the blue light receptors aureochromes in eukaryotes. Planta.

[20] Deng Y., Yao J., Fu G., Guo H., Duan D. (2014). Isolation, expression, and characterization of blue light receptor aureochrome gene from *Saccharina japonica* (laminariales, phaeophyceae). Mar. Biotechnol..

[21] Bowler C., Allen A.E., Badger J.H., Grimwood J., Jabbari K., Kuo A., Maheswari U., Martens C., Maumus F., Otillar R.P. (2008). The *Phaeodactylum* genome reveals the evolutionary history of diatom genomes. Nature.

[22] Serif M., Lepetit B., Weißert K., Kroth P.G., Bartulos R. (2017). A fast and reliable strategy to generate TALEN-mediated gene knockouts in the diatom *Phaeodactylumtricornutum*. Algal Res..

[23] Kroth P.G., Wilhelm C., Kottke T. (2017). An update on aureochromes: Phylogeny–mechanism–function. J. Plant Physiol..

[24] Herrou J., Crosson S. (2011). Function, structure and mechanism of bacterial photosensory LOV proteins. Nat. Rev. Microbiol..

[25] Toyooka T., Hisatomi O., Takahashi F., Kataoka H., Terazima M. (2011). Photoreactions of aureochrome-1. Biophys. J..

[26] Herman E., Kottke T. (2015). Allosterically regulated unfolding of the A′ α helix exposes the dimerization site of the blue-light-sensing aureochrome-LOV domain. Biochemistry.

[27] Heintz U., Schlichting I. (2015). Blue light-induced LOV domain dimerization enhances the affinity of Aureochrome 1a for its target DNA sequence. Elife.

[28] Hisatomi O., Takeuchi K., Zikihara K., Ookubo Y., Nakatani Y., Takahashi F., Kataoka H. (2012). Blue light-induced conformational changes in a light-regulated transcription factor, aureochrome-1. Plant Cell Physiol..

[29] Huysman M.J., Fortunato A.E., Matthijs M., Costa B.S., Vanderhaeghen R., Van D.H., Sachse M., Inzé D., Bowler C., Kroth P.G. (2013). Aureochrome1a-mediated induction of the diatom-specific cyclin dscyc2 controls the onset of cell division in diatoms (*phaeodactylum tricornutum*). Plant Cell.

[30] Volarević S., Stewart M.J., Ledermann B., Zilberman F., Terracciano L., Montini E., Grompe M., Kozma S.C., Thomas G. (2000). Proliferation, but not growth, blocked by conditional deletion of 40S ribosomal protein S6. Science.

[31] Stynen B., Tournu H., Tavernier J., Dijck P.V. (2012). Diversity in genetic in vivo methods for protein-protein interaction studies: From the yeast two-hybrid system to the mammalian split-luciferase system. Microbiol Molecul. Biol. R..

[32] Jacquier A. (2002). Two-hybrid systems — methods and protocols. edited by paul n. macdonald, published by humana press. Biochimie.

[33] Ruvinsky I., Meyuhas O. (2006). Ribosomal protein s6 phosphorylation: From protein synthesis to cell size. Trends Biochem. Sci..

[34] Ruvinsky I., Sharon N., Lerer T., Cohen H., Stolovich R.M., Nir T., Dor Y., Zisman P., Meyuhas O. (2005). Ribosomal protein S6 phosphorylation is a determinant of cell size and glucose homeostasis. Genes Dev..

[35] Enganti R., Cho S.K., Toperzer J.D., Urquidi C.R.A., Cakir O.S., Ray A.P., Abraham P.E., Hettich R.L., Arnim A.G. (2018). Phosphorylation of ribosomal protein RPS6 integrates light signals and circadian clock signals. Front. Plant Sci..

[36] Yao J., Fu W., Wang X., Duan D. (2009). Improved RNA isolation from *Laminaria japonica* Aresch (Laminariaceae, Phaeophyta). J. Appl. Phycol..

[37] Shao Z., Liu F., Li Q., Yao J., Duan D. (2014). Characterization of ribulose-1, 5-bisphosphate carboxylase/oxygenase and transcriptional analysis of its related genes in *Saccharina japonica* (Laminariales, Phaeophyta). Chin. J. Oceanol. Limn..

[38] Zhao W., Li X., Liu W.H., Zhao J., Jin Y.M., Sui T.T. (2014). Construction of high-quality caco-2 three-frame cDNA library and its application to yeast two-hybrid for the human astrovirus protein–protein interaction. J. Virol. Methods..

