# Supplementary material for: The 40S Ribosomal Protein S6 Response to Blue Light by Interaction with SjAUREO in Saccharina japonica
Source: Int J Mol Sci. 2019 May 15;20(10):2414. doi: 10.3390/ijms20102414 (PMC6566693; doi:10.3390/ijms20102414)
Supplement: Supplementary file 1 [file ijms-20-02414-s001.pdf]

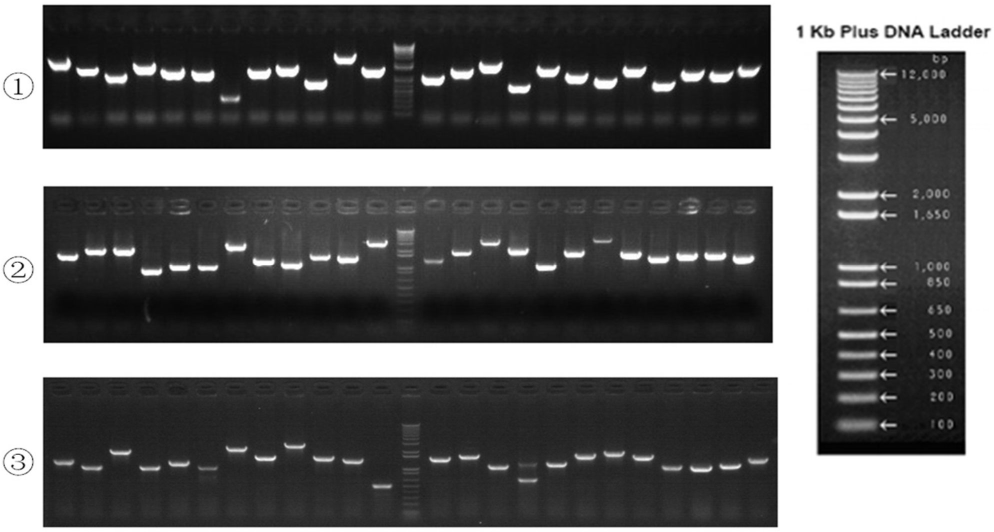

Supplemental Figure 1 Insert detection of three cDNA libraries by agarose gel.

```
1   CATATGTCGGAGCAGCAGAAGCTGGAGAGACGGGAGCGCAACAGAGAGCACGCGAAGCGATCACGAATTCGCAAGAAGTT AUREO in pGBK
1   ---ATGTCGGAGCAGCAGAAGCTGGAGAGACGGGAGCGCAACAGAGAGCACGCGAAGCGATCACGAATTCGCAAGAAGTT AUREO CDS

81  CATGTTGGAATGTCTACAGGAGCAGCTCCTCGCCATGCGGAAGCAGAATATGGCTCTGAGACAGGTGGTGAAAGAGCACA AUREO in pGBK
78  CATGTTGGAATGTCTACAGGAGCAGCTCCTCGCCATGCGGAAGCAGAATATGGCTCTGAGACAGGTGGTGAAAGAGCACA AUREO CDS

161 TGCCCAACGAGGCCGCAACGGTGTTCGAGAAGTGTGTTCATGAGAAGGCCCTTGCGCTCTCTGGCAAGGGCACGGGTTCG AUREO in pGBK
158 TGCCCAACGAGGCCGCAACGGTGTTCGAGAAGTGTGTTCATGAGAAGGCCCTTGCGCTCTCTGGCAAGGGCACGGGTTCG AUREO CDS

241 GCGCAAGCTATCCAATCAGACGACGAGGACGAATCAAAGGAACCGAACTGCCTGTTGCTAGAGCCAGACTTCCAGCTGAT AUREO in pGBK
238 GCGCAAGCTATCCAATCAGACGACGAGGACGAATCAAAGGAACCGAACTGCCTGTTGCTAGAGCCAGACTTCCAGCTGAT AUREO CDS

321 GCAGGCCCTCATGGAGAGCCAGCAGAACTTCACGATCTCAGACCCTAGCATGGCGGACAACCCCATCGTCTACGCGAGCC AUREO in pGBK
318 GCAGGCCCTCATGGAGAGCCAGCAGAACTTCACGATCTCAGACCCTAGCATGGCGGACAACCCCATCGTCTACGCGAGCC AUREO CDS

401 AGGGCTTTTTGACGTTGACGGGCTACACAATCCAGAACGTCATCGGCCGAAACTGCCGCTTCCTGCAGGGGCCTGGAACG AUREO in pGBK
398 AGGGCTTTTTGACGTTGACGGGCTACACAATCCAGAACGTCATCGGCCGAAACTGCCGCTTCCTGCAGGGGCCTGGAACG AUREO CDS

481 GACCCGAGGGCGATCGACATCATCCGTCGAGGGGTGGCCGAGGGGCGAGACACGAGCGTCTGTCTCATGAACTACAAGGC AUREO in pGBK
478 GACCCGAGGGCGATCGACATCATCCGTCGAGGGGTGGCCGAGGGGCGAGACACGAGCGTCTGTCTCATGAACTACAAGGC AUREO CDS

561 GGACGGCACCCCGTTTTGGAACCAGTTTTTCGTTGCCGTACAGGGGCTGCGGGTCGAC                       AUREO in pGBK
558 GGACGGCACCCCGTTTTGGAACCAGTTTTTCGTTGCCGTACAGGGGCTGCGG                             AUREO CDS
```

Supplemental Figure 2 The alignment of AUREO CDS sequence and its sequence in pGBKT7 vector.
